# Alnuctamab, a bivalent B-cell maturation antigen-targeting T cell engager for patients with relapsed or refractory multiple myeloma: results from a phase 1, first-in-human study

**DOI:** 10.1038/s41375-025-02841-x

**Published:** 2026-01-07

**Authors:** Noffar Bar, Thomas Martin, Craig C. Hofmeister, Maria-Victoria Mateos, Markus Hansson, Laura Paris, Swathi Namburi, Paz Ribas, Armando Santoro, Paula Rodriguez-Otero, Maria Creignou, Jinjie Chen, Cong Cao, Brian Kiesel, Allison Gaudy, Ethan G. Thompson, Ye Shen, Samah Zarif, Kevin Hsu, Suresh G. Shelat, Michael R. Burgess, Colin Godwin, Luciano J. Costa

**Affiliations:** 1 Yale University School of Medicine, Yale Cancer Center, New Haven, CT USA; 2 University of California, San Francisco, CA USA; 3 Winship Cancer Institute of Emory University, Atlanta, GA USA; 4 University Hospital of Salamanca, IBSAL, Cancer Research Center-IBMCC (USAL-CSIC), Salamanca, Spain; 5 Skåne University Hospital, Lund, Sweden; 6 Sahlgrenska University Hospital, Göteborg, Sweden; 7 ASST Papa Giovanni XXIII, Bergamo, Italy; 8 Swedish Cancer Institute, Swedish Medical Center, Seattle, WA USA; 9 Hospital Universitario Dr Peset Aleixandre, Valencia, Spain; 10 Humanitas University, Pieve Emanuele, Milan, Italy; 11 IRCCS Humanitas Research Hospital, Humanitas Cancer Center, Rozzano, Milan, Italy; 12 Cancer Center Clínica Universidad de Navarra (CCUN), CIMA, CIBERONC, IDISNA, Pamplona, Spain; 13 Phase 1 Unit, Department of Clinical Cancer Studies, Karolinska University Hospital, Stockholm, Sweden; 14 Bristol Myers Squibb, Princeton, NJ USA; 15 Bristol Myers Squibb, Boudry, Switzerland; 16 University of Alabama at Birmingham, Birmingham, AL USA

**Keywords:** Myeloma, Myeloma

## Abstract

B-cell maturation antigen (BCMA)-targeting therapies provide a new approach to treating multiple myeloma (MM). Alnuctamab (ALNUC) is a 2 + 1 immunoglobulin G1-based bispecific antibody binding BCMA and CD3ε receptors on myeloma and T cells, respectively. CC-93269-MM-001 is a first-in-human, phase 1 dose escalation/expansion study investigating ALNUC in relapsed/refractory MM. Patients had ≥3 prior regimens, disease progression ≤60 days of last regimen, and were BCMA-directed therapy-naïve. ALNUC was administered intravenously (IV) and subcutaneously (SC); however, SC was selected for further evaluation due to the more favorable safety profile. Ninety-five patients received ALNUC SC; at data cutoff, 44.2% remained on treatment and median follow-up was 8.0 months. The recommended phase 2 dose was 30 mg. The most common treatment emergent adverse events (any grade/grade 3/4) were CRS (57.9%/0%), and neutropenia (53.7%/43.2%). Infections were also frequent (64.2%/14.7%). ORR was 58.9% for all ALNUC SC-treated patients and 71.4% for the 30-mg cohort; 47/95 (49.5%) were measurable residual disease (MRD) negative. Overall, the safety and efficacy of ALNUC SC were comparable to other BCMA-targeted therapies. These results support improved safety of SC versus IV, and corroborate a step-up dosing strategy to mitigate CRS. Importantly, a schedule that de-intensifies over time provides favorable toxicity that may be applicable to other bispecific engagers.

## Introduction

Multiple myeloma (MM) is characterized by multiple remissions and relapses, with prognosis worsening with each relapse [1–3]. Several B-cell maturation antigen (BCMA)-targeting therapies have been approved for use in patients with MM, some of which have demonstrated significant life-prolonging efficacy for this patient population [2–7]. Approved BCMA-targeted T-cell redirecting therapies include chimeric antigen receptor (CAR) T cell therapies (idecabtagene vicleucel and ciltacabtagene autoleucel) and bispecific antibodies (teclistamab, elranatamab, and linvoseltamab). These treatments have unique toxicities, including cytokine release syndrome (CRS) and neurotoxicity, which require special management [2–8]. Due in part to the suppressed levels of normal plasma cells and B cells, in response to these therapies, the management of infections is also an important consideration in patients with relapsed or refractory MM (RRMM) who are receiving BCMA-targeted agents [2–7].

Alnuctamab (ALNUC) differs from other BCMA-targeting bispecific antibodies in its structure. It is a humanized 2 + 1 immunoglobulin (Ig) G1-based bispecific antibody, which binds bivalently to BCMA on myeloma cells and monovalently to CD3ε on T cells [9, 10]. ALNUC binding is highly specific, with high affinity and avidity for BCMA and low affinity for CD3ε, and promotes strong CD3+ T-cell/myeloma cell interactions [9, 10]. Bivalent binding enhances tumor-antigen avidity, while monovalent binding to CD3ε, with a dissociation constant of 70–100 nM, is important for limiting nonspecific T-cell activation in the absence of BCMA binding [9, 10].

ALNUC has demonstrated profound antitumor activity in preclinical models of RRMM, and can be given intravenously (IV) or subcutaneously (SC) [11, 12]. CC-93269-MM-001 (NCT03486067) is a first-in-human, open-label phase 1 dose escalation and expansion study investigating ALNUC in patients with RRMM. Here, we report the safety and efficacy outcomes of ALNUC IV and SC, along with pharmacokinetic (PK) data for dose determination and translational findings that provide insight into predictive markers of response and adverse events (AEs).

## Methods

### Patients

This was a first-in-human, open-label phase 1 dose escalation and expansion clinical study of ALNUC in patients with RRMM. Patients were required to have had RRMM treated with ≥3 prior regimens, including a proteasome inhibitor, an immunomodulatory (IMiD) drug, and an anti-CD38 antibody. Additionally, patients must have had disease progression within 60 days of their last regimen. Patients with a history of prior BCMA-directed therapy were not eligible, except for a small cohort (*n* = 6) of patients treated with ALNUC IV who had previously received BCMA-targeting therapy.

### Study intervention

ALNUC (0.15–10 mg) IV was given weekly (QW) in cycle (C) 1–3 (after step-up doses, if used), every 2 weeks (Q2W) in C4–6, and every 4 weeks (Q4W) in C7+ (28-day cycles). ALNUC SC administration included 3 mg and 6 mg step-up doses on C1, day (D) 1 and C1D4, respectively, followed by target doses of ALNUC SC 10–60 mg on D8, D15, and D22 of C1, QW in C2–3, Q2W in C4–6, and Q4W after 6 months. The target doses of ALNUC SC 10, 15, 30, and 60 mg were evaluated in the dose escalation phase; doses of 10, 30, and 60 mg were evaluated in the dose expansion phase.

Premedication with dexamethasone for CRS was required prior to administration of the step-up doses, after any dose increase, and after an infusion interruption of > 2 weeks during C1–6.

### Infection mitigation strategies

Enrolled patients were monitored for hypogammaglobulinemia and infection, including opportunistic infections. Administration of ALNUC was postponed in patients with suspected, active, or uncontrolled infections, until the infection resolved. To prevent hypogammaglobulinemia, enrolled patients with IgG <400 mg/dL were recommended to receive IV Ig replacement to maintain an IgG level ≥400 mg/dL. Further infection mitigation strategies were implemented in patients receiving ALNUC IV after March 2020 in response to grade ≥3 infections. These mitigation strategies were subsequently applied to all patients receiving ALNUC SC (enrollment years, 2021–2024). These included delayed dosing for patients with respiratory viruses; prophylaxis against bacteria (e.g., levofloxacin; C1–3 and during grade ≥3 neutropenia), *Pneumocystis jirovecii* pneumonia (e.g., trimethoprim-sulfamethoxazole; all cycles), and herpes simplex virus/varicella zoster virus (e.g., acyclovir; all cycles).

### Study endpoints

The primary endpoints were safety, including AEs, dose-limiting toxicities, non-tolerated doses (NTDs), and maximum tolerated doses (MTDs). Secondary endpoints included efficacy, overall response rate (ORR), time to response (TTR), duration of response (DOR), progression-free survival (PFS), overall survival (OS), and PK. Exploratory endpoints included measurable residual disease (MRD) and translational medicine assessments. Grading of AEs was performed according to Common Terminology Criteria for AEs v4.03, and the grading for CRS was according to Lee et al. [13, 14]. Responses were investigator-assessed according to the International Myeloma Working Group criteria. Lastly, PK, immunogenicity, and exposure-response analysis were fully characterized previously [15]. MRD was evaluated by EuroFlow, and samples with a minimum sensitivity of 10^−5^ by flow cytometry were considered MRD-negative.

The study was conducted in accordance with Good Clinical Practice, as described in International Conference on Harmonisation Guideline E6, and the general ethical principles outlined in the Declaration of Helsinki. The study was approved by the institutional review boards/ethics committees of the participating sites, and informed consent was obtained from the accrued patients. All authors had access to the data, contributed to the analysis, and confirmed the accuracy and completeness of the data as well as adherence to the protocol.

### Cytokine profiling and cytogenetic assessment

Cytokines and immune-related soluble factors were measured from plasma samples using Luminex multiplex platforms HCD8 MAG-17K (Millipore) and LXSAHM-8 (R&D Systems). Bone marrow aspirate samples were collected at baseline for the assessment of cytogenetic abnormalities. High-risk (HR) cytogenetic abnormality was defined as having at least one of the following at any detectable level by fluorescence in situ hybridization: 17p deletion, t(4:14), t(14:16), or 1q21 amplification.

### Statistical analysis

Approximately 3–6 patients per dosing cohort in the dose escalation phase were used to determine the MTD and NTD of both the first dose and subsequent doses of ALNUC. Sample sizes in the dose expansion phase were determined based on Bayesian dual-criterion approach and clinical considerations traditionally used for exploratory studies of this kind.

Safety was assessed in all patients who received ≥1 dose of ALNUC. Point estimates and two-sided, 95% confidence intervals (CIs) of ORR were reported. Kaplan–Meier (KM) survival analyses were performed for DOR, OS, and PFS; if evaluable, the median time-to-event data were presented with 95% CIs.

## Results

### Patients

A total of 70 patients were treated with ALNUC IV with doses ranging from 0.15 to 10 mg and a median follow-up of 8.0 months. Due to safety signals including grade ≥3 CRS (7.1%) and one case of grade 5 CRS, as well as a suboptimal ORR of 40.0%, the study pivoted to ALNUC SC. Additional data on patients treated with ALNUC IV can be found in the data supplement. In total, 95 patients received ALNUC SC in the dose escalation and dose expansion phases, at target doses of 10 mg (*n* = 25), 15 mg (*n* = 4), 30 mg (*n* = 49), or 60 mg (*n* = 17). The median follow-up was 11.8 months. Baseline characteristics and prior treatment history of patients treated with ALNUC SC and IV are shown in Table 1 and Supplementary Table 1, respectively. For all target doses of ALNUC SC, patients had a median of 4 (range, 3–14) prior lines of treatment prior to study enrollment, 23 (24.2%) patients had extramedullary disease (EMD), and 24 (25.3%) patients had a HR cytogenetic profile.

**Table 1:** Baseline characteristics of the ALNUC SC cohort.

Patient characteristics	All target doses (*n* = 95)	30-mg target dose (*n* = 49)
Median age, years (range)	64 (36–85)	65 (40–85)
Sex, *n* (%)		
Male	52 (54.7)	28 (57.1)
Female	43 (45.3)	21 (42.9)
Ethnicity, *n* (%)		
White	70 (73.7)	35 (71.4)
African American/Black	13 (13.7)	6 (12.2)
Asian	2 (2.1)	1 (2)
Not reported	9 (9.5)	6 (12.2)
ECOG performance status, *n* (%)		
0	38 (40)	19 (38.8)
1	57 (60)	30 (61.2)
Median time since initial diagnosis, years (range)	5.79 (0.7–24.2)	5.74 (0.7–20.2)
Derived ISS stage^a^		
I	41 (43.2)	24 (49.0)
II	39 (41.1)	18 (36.7)
III	15 (15.8)	7 (14.3)
High-risk cytogenetics, *n* (%)^b^	24 (25.3)	15 (30.6)
EMP^c^, *n* ‘yes’ (%)	23 (24.2)	11 (22.4)
Median BMPC, % (range)		
Aspirate	4 (0–97)	2 (0–74)
Biopsy	10 (0–95)	4 (0–90)
Prior treatment history	All target doses (*n* = 95)	30-mg target dose (*n* = 49)
Median no. of prior therapies, *n* (range)	4 (3–14)	4 (3–14)
Refractory to last therapy, *n* (%)	92 (96.8)	48 (98)
Prior anti-CD38, *n* (%)	95 (100)	49 (100)
Prior PI, *n* (%)	95 (100)	49 (100)
Prior IMiD^TM^, *n* (%)	95 (100)	49 (100)
Prior triple-class–exposed^d^, *n* (%)	94 (98.9)	48 (98)
Prior penta-drug–exposed^e^, *n* (%)	71 (74.7)	34 (69.4)
Refractory, *n* (%)		
Triple	61 (64.2)	31 (63.3)
Penta	19 (20)	10 (20.4)
PI	73 (76.8)	38 (77.6)
IMiD	77 (81.1)	41 (83.7)
CD38	88 (92.6)	46 (93.9)
Stem cell transplantation, *n* (%)		
Autologous	80 (84.2)	40 (81.6)
Allogeneic	4 (4.2)	1 (2.0)

*ALNUC* alnuctamab, β*2m* β2-microglobulin, *BMPC* bone marrow plasma cells, *ECOG* Eastern Cooperative Oncology Group, *EMP* extramedullary plasmacytoma, *IMiD* immunomodulatory imide drug, *ISS* international staging system, *PI* proteasome inhibitor, *SC* subcutaneous.

^a^Based on baseline β2m (central lab) and albumin (local lab).

^b^At least one of the following: 17p del, t(4:14), t(14:16), and 1q21 amp in central lab specimen.

^c^EMPs were defined as true soft tissue plasmacytomas; paraosseous plasmacytomas were not included in the EMP definition.

^d^Exposed/refractory to a PI, an IMiD agent (including thalidomide), and an anti-CD38 antibody.

^e^Exposed/refractory to two PIs (bortezomib, carfilzomib), two IMiD agents (any two of pomalidomide, lenalidomide, and thalidomide), and an anti-CD38 antibody.

At the data cutoff (April 2, 2024), a total of 53 (55.8%) patients treated with ALNUC SC discontinued treatment due to progressive disease (*n* = 40), withdrawal (*n* = 4), lack of efficacy (*n* = 4), AEs (*n* = 3), or death (*n* = 2) (Supplementary Fig. 1A), while 42 (44.2%) patients in the SC cohort remained on treatment, including 28 (57.1%) patients treated at the 30-mg target dose (Supplementary Fig. 1B), which was selected as the recommended phase 2 dose.

### Safety

TEAEs for the ALNUC IV cohort are summarized in Supplementary Table 2.

In the ALNUC SC treatment cohort, any-grade and grade 3/4 treatment-emergent AEs (TEAEs) were reported in 94 (98.9%) and 71 (74.7%) patients, respectively (Table 2). The most common TEAEs according to system and organ class included blood and lymphatic system disorders (occurring in 73 [76.8%] patients) and infections and infestations (occurring in 61 [64.2%] patients). Neutropenia was the most common grade 3/4 TEAE occurring in 41 (43.2%) patients. The median time to resolution of the first occurrence of grade 3/4 neutropenia (to grade ≤ 2) was 1.1 weeks (range, 0.3–5.1).

**Table 2:** TEAEs reported in ≥ 10% of patients treated with ALNUC SC.

TEAEs, *n* (%)	All target doses (*n* = 95)		30-mg target dose (*n* = 49)	
	All grade	Grade 3/4	All grade	Grade 3/4
Patients with ≥ 1 TEAE	94 (98.9)	71 (74.7)	49 (100.0)	34 (69.4)
Blood and lymphatic system disorders	73 (76.8)	59 (62.1)	35 (71.4)	29 (59.2)
Neutropenia	51 (53.7)	41 (43.2)	23 (46.9)	20 (40.8)
Anemia	44 (46.3)	22 (23.2)	19 (38.8)	8 (16.3)
Thrombocytopenia	34 (35.8)	14 (14.7)	13 (26.5)	6 (12.2)
Lymphopenia	15 (15.8)	13 (13.7)	6 (12.2)	5 (10.2)
Leukopenia	10 (10.5)	5 (5.3)	3 (6.1)	2 (4.1)
Non-hematologic TEAEs				
Infections and infestations	61 (64.2)	14 (14.7)	34 (69.4)	5 (10.2)
CRS	55 (57.9)	0 (0)	28 (57.1)	0 (0)
Headache	25 (26.3)	0 (0)	16 (32.7)	0 (0)
Back pain	21 (22.1)	1 (1.1)	13 (26.5)	1 (2.0)
Fatigue	22 (23.2)	0 (0)	12 (24.5)	0 (0)
Diarrhea	28 (29.5)	1 (1.1)	14 (28.6)	0 (0)
Arthralgia	19 (20.0)	1 (1.1)	12 (24.5)	0 (0)
Insomnia	18 (18.9)	0 (0)	10 (20.4)	0 (0)
Cough	20 (21.1)	0 (0)	11 (22.4)	0 (0)
Hypogammaglobulinemia	19 (20.0)	0 (0)	7 (14.3)	0 (0)
Nausea	22 (23.2)	0 (0)	14 (28.6)	0 (0)
Hypomagnesemia	17 (17.9)	1 (1.1)	7 (14.3)	1 (2.0)
Constipation	14 (14.7)	0 (0)	8 (16.3)	0 (0)
ALT increase	14 (14.7)	5 (5.3)	7 (14.3)	1 (2.0)
AST increase	11 (11.6)	2 (2.1)	4 (8.2)	0 (0)
Pyrexia	13 (13.7)	0 (0)	4 (8.2)	0 (0)
Peripheral edema	10 (10.5)	0 (0)	5 (10.2)	0 (0)
Hypocalcemia	13 (13.7)	1 (1.1)	8 (16.3)	1 (2.0)
Hypokalemia	10 (10.5)	0 (0)	5 (10.2)	0 (0)

*ALNUC* alnuctamab, *ALT* alanine aminotransferase, *AST* aspartate aminotransferase, *CRS* cytokine release syndrome, *TEAE* treatment-emergent adverse event, *SC* subcutaneous.

Of patients treated with ALNUC SC across all target doses, 36 (37.9%) experienced a serious TEAE, with 18 (36.7%) occurring in patients treated at the 30-mg target dose. Among hematologic disorders, the only serious TEAE was febrile neutropenia, which was reported in 4 (4.2%) patients treated across all target doses, and 2 (4.1%) patients treated at the 30-mg target dose (Supplementary Table 3). Among non-hematologic TEAEs, general physical health deterioration was the most common serious TEAE for both the overall target dose group (occurring in 5 [5.3%] patients) and 30-mg target dose group (occurring in 3 [6.1%] patients).

In the SC treatment cohorts, CRS was reported in 55 (57.9%) patients, including 28 (57.1%) patients treated at the 30-mg target dose (Table 2). The median time to first onset of CRS from first dose was 3.0 days (range, 1–20). All cases were grade 1 or 2 events (Table 3). CRS was most common with the first step-up dose, where 38 (40.0%) patients experienced CRS of any grade, and less frequent and severe with the second and third doses, 17 (18.1%) and 18 (20.2%) patients, respectively. The median duration of CRS in patients treated with ALNUC SC, was 2 days in the overall cohort (range, 1–11) and 1 day (range, 1–9) for patients treated with the 30-mg target dose (Table 3 and Supplementary Table 4). Thirty-two (58.2%) patients with CRS, including 16 (57.1%) in the 30-mg target dose cohort, were treated with tocilizumab (Table 3). Investigator-assessed neurotoxicity consistent with immune effector cell-associated neurotoxicity syndrome was reported in 2 patients (grade 1) in the ALNUC SC cohort, but none were reported in the 30 mg target dose cohort.

**Table 3:** CRS: prevalence, onset, and duration for ALNUC SC.

Patient characteristics	All target doses (*n* = 95)	30-mg target dose (*n* = 49)
Patients with at least 1 CRS, *n* (%)	55 (57.9)	28 (57.1)
Maximum reported CRS grade, *n* (%)		
Grade 1	46 (48.4)	25 (51.0)
Grade 2	9 (9.5)	3 (6.1)
Grade ≥ 3	0	0
Median time to first onset of CRS from first dose, days (range)	3 (1–20)	3 (2–10)
Median duration of CRS, days (range)	2 (1–11)	1 (1–9)
CRS medication in patients with CRS, *n*/*N* (%)		
Tocilizumab	32/55 (58.2)	16/28 (57.1)
Corticosteroid for systemic use	19/55 (34.5)	10/28 (35.7)

*ALNUC* alnuctamab, *CRS* cytokine release syndrome, *SC* subcutaneous.

Sixty-one (64.2%) patients developed infections, the majority of which were low-grade (Table 2). The most frequent types of infections were COVID-19 (*n* = 22; 23.2%) and upper respiratory tract infections (*n* = 14; 14.7%), including rhinovirus infection (*n* = 13; 13.7%). Grade 3/4 infections were reported in 14 (14.7%) patients (Supplementary Table 5), and 1 (1%) patient experienced a grade 5 infection (influenza). Opportunistic infections included asymptomatic cytomegalovirus infection reactivation in 4 (4%) patients and grade 2 fungal esophagitis in 1 (1%) patient. At least one dose of intravenous immune globulins was administered or continued from baseline in 49 (51.6%) patients in the overall SC cohort and 27 (55.1%) patients in the 30-mg target dose cohort.

In total, 23 (24.2%) patients treated with ALNUC SC died. Most of the deaths occurred after the safety follow-up period (>35 days after last dose), including 10 (20.4%) patients in the 30-mg target dose cohort (Supplementary Table 6). Of the 23 deaths, 13 were attributed to disease progression, 6 were caused by an AE, 1 was due to another cause, and 3 were of unknown cause. During the treatment period and the safety follow-up period (≤35 days after the last dose of ALNUC SC, based on the projected half-life using nonclinical data), 7 (7.4%) patients in the overall cohort and 3 (6.1%) patients in the 30-mg target dose cohorts died. Three deaths in the overall cohort occurring within the safety follow-up period were due to an TEAE (respiratory infection with influenza A virus, severe bleeding, and cerebral hemorrhage; only the cerebral hemorrhage was reported as related to ALNUC), and the remaining deaths were attributed to disease progression (*n* = 3), or were of unknown cause (*n* = 1).

### Efficacy

**Fig. 1:**
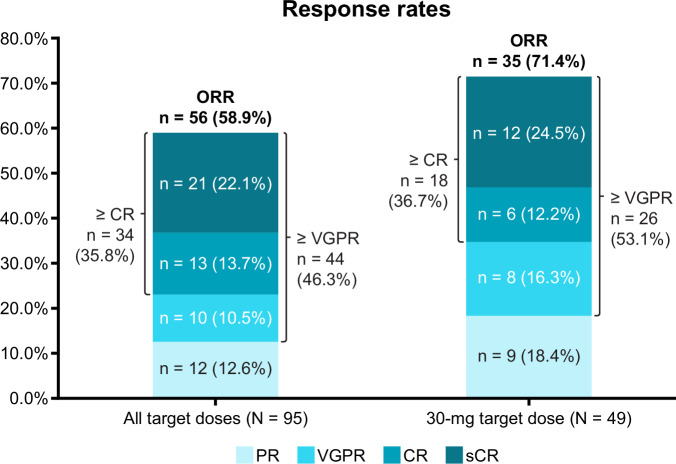
Patient response rates. CR complete response, ORR objective response rate, PR partial response, sCR stringent complete response, VGPR very good partial response.

**Fig. 2:**
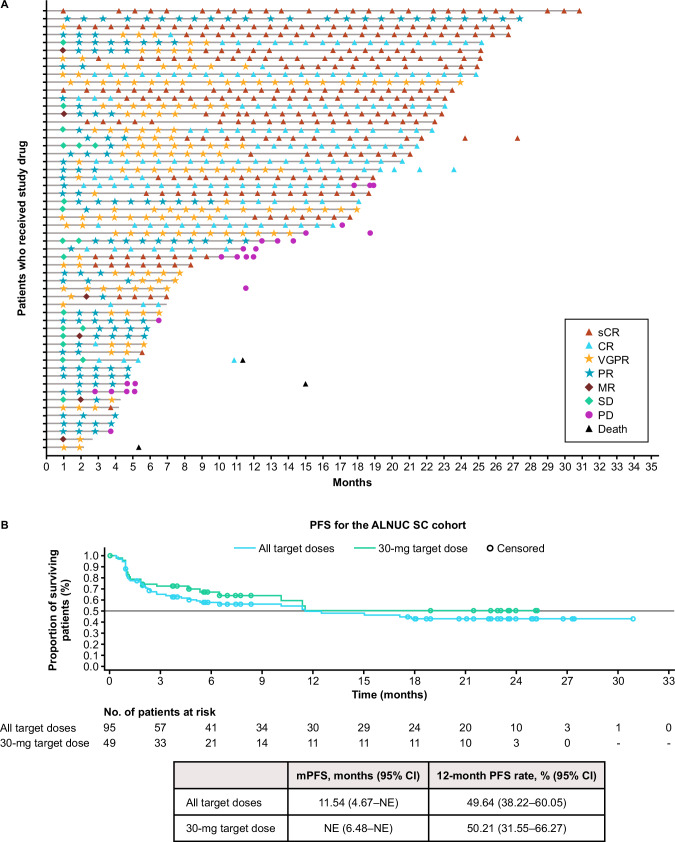
Efficacy of ALNUC SC. Patient responses over time (**A**) and PFS for the ALNUC SC cohort (**B**). ALNUC alnuctamab, CI confidence interval, CR complete response, mPFS median progression-free survival, MR minimal response, NE not evaluable, PFS progression-free survival, PD progressive disease, PR partial response, SC subcutaneous, sCR stringent complete response, SD stable disease, VGPR very good partial response.

The ORR (percentage of patients achieving partial response or better) was 58.9% (*n* = 56) in the 95 patients treated with ALNUC SC, including 71.4% (*n* = 35) in the 49 patients treated with ALNUC 30 mg SC. The percentage of patients achieving at least a complete response (CR) was 35.8% (*n* = 34) and 36.7% (*n* = 18) for the all-target dose and 30-mg target dose cohorts, respectively (Fig. 1). Subgroup analyses showed that at the 30-mg dose, a numerically higher ORR was observed in patients with international staging system (ISS) stage I/II disease (34/42; 81.0%) versus ISS stage III disease (1/7; 14.3%); in patients without penta-refractory disease (19/24; 79.2%) versus with (5/10; 50.0%); and lower baseline (≤the SC cohort median of 161 ng/mL) soluble BCMA (sBCMA) (13/14; 92.9%) versus higher (5/13; 38.5%) (Supplementary Fig. 2). The ORR for patients with baseline EMD was numerically lower (7/11; 63.6%) than that of patients without EMD at baseline (28/38; 73.7%). Responses deepened over time and were durable (Fig. 2A). The median TTR was 4.5 weeks (range, 4.0–17.4) and 4.6 weeks (range, 4.0–16.3) for the all-target dose and 30-mg target dose, respectively. At the time of the data cutoff, the median DOR was not reached and the median follow-up was 11.8 months. The median PFS was 11.5 months (95% CI, 4.7–not evaluable) across doses and not evaluable (95% CI, 6.5–not evaluable) at the 30-mg target dose; 12-month PFS rates were 49.6 (95% CI, 38.2–60.1) and 50.2 (95% CI, 31.6–66.3), respectively (Fig. 2B). Median OS was not reached across doses or at the 30-mg target dose (Supplementary Fig. 3).

**Fig. 3:**
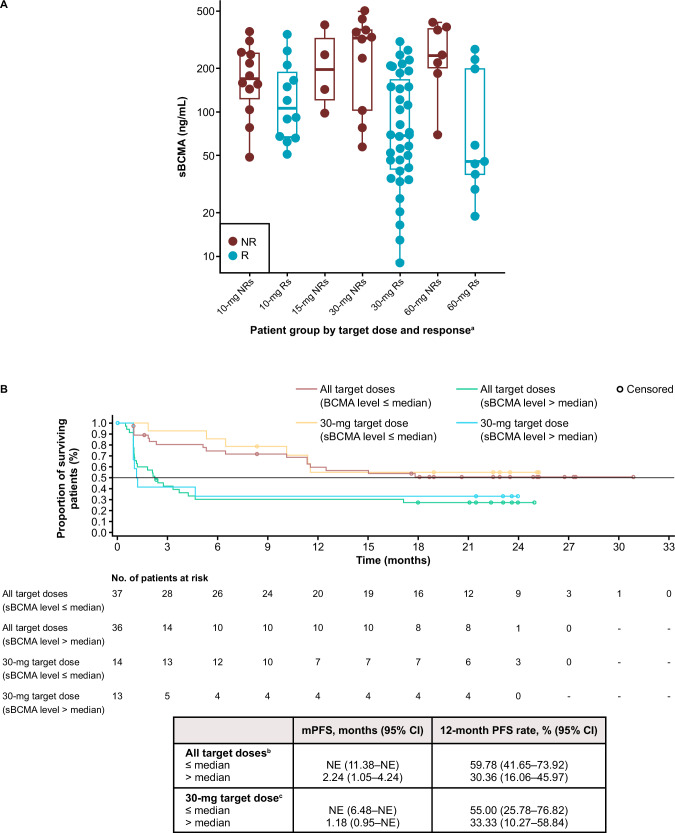
Association of pre-treatment sBCMA and efficacy. Comparison of pre-treatment sBCMA levels between non-responders and responders in each target dose cohort (**A**), and PFS by baseline median sBCMA level (**B**). ^a^*p* = 0.000002 when comparing Rs versus NRs in all patients receiving ALNUC SC; *p* = 0.0009 when comparing Rs versus NRs in the ALNUC 3-, 6-, and 30-mg cohorts. ^b^Median baseline sBCMA = 161 ng/mL. ^c^Median baseline sBCMA = 113 ng/mL. ALNUC alnuctamab, mPFS median progression-free survival, NR non-responder, PFS progression-free survival, R responder, sBCMA soluble B-cell maturation antigen, SC subcutaneous.

Lower sBCMA baseline levels were seen in responders versus non-responders across target doses, as shown by individual patient data (Fig. 3A). In addition, KM analysis indicated that lower baseline median sBCMA levels were associated with longer PFS and higher 12-month survival rates across both cohorts (Fig. 3B).

Efficacy results for the ALNUC IV cohort are summarized in Supplementary Table 7.

A total of 49 (51.6%) patients treated with ALNUC SC who achieved a response had samples evaluable for MRD. Across all target doses, 47/95 (49.5%) patients treated with ALNUC SC were MRD-negative at, or after, C2D1 (Table 4). Of the 49 patients treated at the 30-mg target dose, 28 (57.1%) patients were MRD-negative. In patients with CR or better, the MRD-negative response was 88.2% (30/34) in all SC-treated patients and 94.4% (17/18) in patients who received the 30-mg target dose.

**Table 4:** MRD rate for ALNUC SC.

	All target doses (*n* = 95)	30-mg target dose (*n* = 49)
Responders (PR or better), *n* (% of safety population)	56/95 (58.9)	35/49 (71.4)
Responders with evaluable MRD post ALNUC treatment, *n* (% of safety population)	49/95 (51.6)	30/49 (61.2)
Responders testing MRD-negative post ALNUC treatment, *n*/*N* (% of responders with evaluable MRD results)	47/49 (95.9)	28/30 (93.3)
Responders testing MRD-negative post ALNUC treatment, *n*/*N* (% of total)	47/95 (49.5)	28/49 (57.1)
sCR + CR, *n* (% of safety population)	34/95 (35.8)	18/49 (36.7)
sCR + CR with evaluable MRD post ALNUC treatment, *n* (% of safety population)	34/95 (35.8)	18/49 (36.7)
sCR + CR and MRD-negative post ALNUC treatment,^a^ *n*/*N* (% of sCR + CR with evaluable MRD results)	30/34 (88.2)	17/18 (94.4)
sCR + CR testing MRD-negative post ALNUC treatment (% of total)	30/95 (31.6)	17/49 (34.7)

^a^Counted if the MRD-negative finding occurred within 3 months before or any time after CR/sCR response.

*ALNUC* alnuctamab, *CR* complete response, *MRD* minimal residual disease, *PR* partial response, *sCR* stringent complete response, *SC* subcutaneous.

### Cytokine profiling

**Fig. 4:**
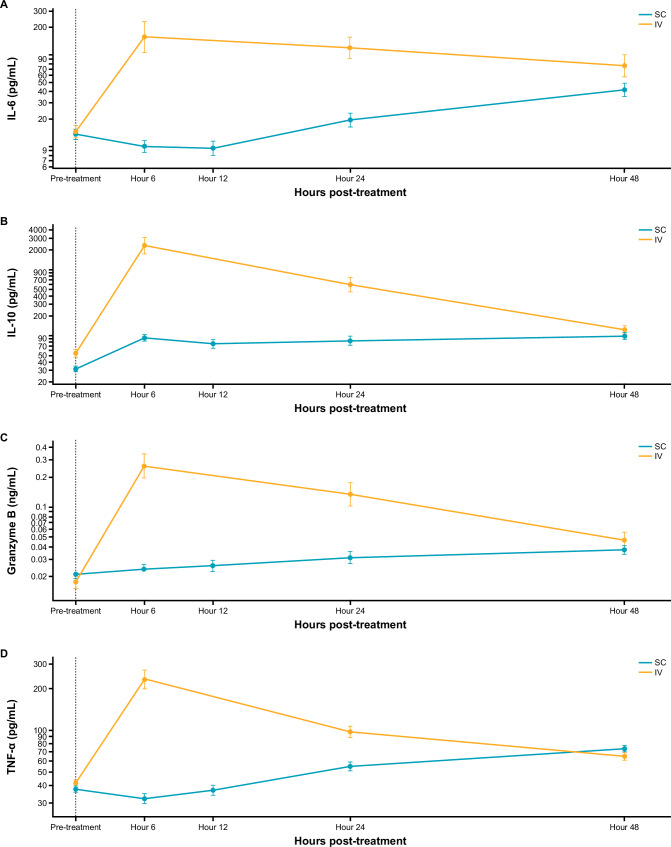
Pattern of cytokines change in response to IV and SC administration of ALNUC. Induction of cytokines IL-6 (**A**), IL-10 (**B**), granzyme B (**C**), and TNFα (**D**) in ALNUC SC versus IV with a 3-mg starting dose after first dose. Each data point represents the geometric mean ± the SEM. ALNUC alnuctamab, IL interleukin, IV intravenous, SC subcutaneous, SEM standard error of the mean, TNF-α tumor necrosis factor alpha.

Inflammatory cytokines and other soluble factors indicative of T-cell–mediated antitumor response were measured prior to, or following administration of ALNUC IV or ALNUC SC at specified time points throughout the treatment period. Plasma levels of interleukin (IL)-6, IL-10, granzyme B, and tumor necrosis factor alpha (TNF-α) over time are shown in Fig. 4. The induction of soluble factors was delayed in ALNUC SC, peaking at 48 h versus 6 h with ALNUC IV. Within the SC cohort, increased target doses of ALNUC resulted in numerically higher levels of secretion of IL-10, TNF-α, and granzyme B, but not IL-6, with induction of those factors peaking at 24–48 h post-treatment (Supplementary Fig. 4).

The induction of selected soluble factors that correlate with T-cell and immune activation (e.g., soluble CD137, granzyme B, and IL-10) after the first step-up dose was higher in responders in the 30-mg ALNUC SC target dose cohort compared with non-responders. Levels of IL-6, one of the most significantly elevated cytokines during CRS, were similar between responders and non-responders in the 30-mg cohorts. Angiopoietin-2, whose serum level has been shown to correlate with RRMM disease progression [16], showed elevated levels at baseline and during ALNUC SC treatment in non-responders (Supplementary Fig. 5).

Responders from SC cohorts achieved deep and durable peripheral B-cell clearance while non-responders only achieved minimal B-cell clearance (Supplementary Fig. 6).

### Pharmacokinetics

PK, immunogenicity, and exposure-response analysis were fully characterized previously [15]. ALNUC PK was linear across the dose range tested, with minimal clinical impact of sBCMA and a half-life of ~14 days. Immunogenicity incidence was low after IV and SC administration. Exposure-response analysis after SC administration demonstrated a significant relationship between increased exposure (area under the curve over the first cycle) and efficacy (ORR), although efficacy approached a plateau at exposures associated with the 30-mg dose. Additionally, a significant relationship was found between increasing maximum serum concentration (C_max_) and any-grade CRS following the first ALNUC SC dose, but no correlation was found after subsequent step-up doses. No exposure-safety relationships were identified concerning hematologic or infection endpoints [15].

## Discussion

We conducted a first-in-human, phase 1, open-label, dose-finding study of ALNUC, a BCMA-targeted therapy, in patients with RRMM treated with ≥ 3 prior lines of therapy. ALNUC can be delivered by IV or SC; here, we show that SC delivery had a favorable safety profile and led to deep and durable responses with a high rate of MRD-negativity. Compared with IV administration, ALNUC SC resulted in lower rates of CRS and reduced secretion of inflammatory cytokines and other soluble factors. The improvement in CRS rates gained from switching IV dosing of ALNUC to SC was supported mechanistically by reduced induction of pro-inflammatory cytokines, including IL-6, IL-10, granzyme B, and TNF [17]. These cytokines reflect immune cell activation and are associated with clinical symptoms of fever, hypoxia, and hypotension. Reductions in pro-inflammatory cytokines aligned with reductions of C_max_ after SC administration. Although concentrations were lower after the first dose, the immune stimulatory effect at the 3-mg target dose was sufficient to escalate to target doses that were not reached after IV dosing.

BCMA-targeting bispecific antibodies are associated with a higher infection risk than non-BMCA–targeting bispecific antibodies [18]. Infection-related complications in patients with RRMM treated with BMCA bispecific antibodies typically occur early after starting treatment and persist over time while on continuous therapy, compared to the reduction of severe infections seen with CAR T cell therapies after 100 days [19]. Among the 61 (64.2%) patients who developed infections, the majority of infections were low-grade; 14.7% were grade 3/4 infections, and 1% was a grade 5 infection. The reduction of the dosing frequency of ALNUC in later cycles and the infection mitigation strategies, including mandatory prophylaxis against bacteria and other pathogens and recommended IV Ig replacement for patients with IgG < 400 mg/dL (see Methods section), may have lowered infection rates in later cycles (Supplementary Fig. 7). While the infection rates were comparable to those of other BCMA-targeted bispecific antibodies, grade 3/4 infections were numerically lower at 14.7% compared with 44.8% with teclistamab after a median follow-up of 14.1 months, 27.3% with elranatamab after a median follow-up of 12.0 months, and 35.9% with linvoseltamab after a median follow-up of 14 months. [7, 8, 20].

ALNUC SC was well tolerated and had a favorable efficacy profile compared with other approved BCMA-targeted therapies, although the limitations of cross-trial comparisons should be considered [3, 5–7]. At the 30-mg target dose, ALNUC SC was associated with low rates of CRS, infections, and neurotoxicity relative to teclistamab, elranatamab, linvoseltamab, and CAR T cell therapies. ORR/CR rates observed with ALNUC 30 mg SC (71.4%/48.6%) compared favorably to those reported for teclistamab (63%/39%) and elranatamab (61%/35%) and were similar to those reported for linvoseltamab (71%/50%). Importantly, many patients remained progression-free beyond 6 months, despite the change in dosing frequency to Q4W. This suggests that early disease control is maintained despite switching to a less intensive schedule, which is likely associated with a positive impact on safety and dosing convenience. Furthermore, recent translational analyses have demonstrated that BCMA-targeted antibody treatment can result in the development of BCMA mutations, which reduce myeloma cell sensitivity to teclistamab and elranatamab but not alnuctamab [21]. This suggests that alnuctamab may be less susceptible to BCMA resistance mutations as a relapse mechanism and could translate into favorable DOR with additional follow-up.

Our comprehensive cytokine profiling supports the greater safety of the SC route and corroborates the use and scheduling of step-up dosing. Importantly, the schedule of administration that de-intensified over time provided a favorable toxicity profile, particularly regarding infections, and an excellent DOR.

The development of ALNUC in MM has since been terminated by the sponsor, a strategic decision that was not based on any patient safety concerns or issues associated with its use. The efficacy, safety, and translational learnings from this program may inform the development of other T-cell redirecting therapies in MM.

In conclusion, this study highlights the potential of an SC-administered, BCMA-targeted therapy, demonstrating a convenient Q4W administration schedule, a favorable safety profile, and low rates of severe infection and CRS while maintaining efficacy in patients with RRMM.

## Supplementary information

Supplement

## Data Availability

Data may be obtained from a third party and are not publicly available. Bristol Myers Squibb will honor legitimate requests for clinical trial data from qualified researchers with a clearly defined scientific objective. Data sharing requests will be considered for Phase II-IV interventional clinical trials that completed on or after January 1, 2008. In addition, primary results must have been published in peer-reviewed journals and the medicines or indications approved in the US, EU, and other designated markets. Sharing is also subject to protection of patient privacy and respect for the patient’s informed consent. Data considered for sharing may include non-identifiable patient-level and study-level clinical trial data, full clinical study reports, and protocols. Requests to access clinical trial data may be submitted using the enquiry form at https://vivli.org/ourmember/bristol-myers-squibb/. (see https://www.icmje.org/recommendations/browse/publishing-and-editorial-issues/clinical-trial-registration.html).

## References

[1] Ramasamy K, Gay F, Weisel K, Zweegman S, Mateos MV, Richardson P. Improving outcomes for patients with relapsed multiple myeloma: challenges and considerations of current and emerging treatment options. Blood Rev. 2021;49:100808. 33863601 10.1016/j.blre.2021.100808

[2] Munshi NC, Anderson LD Jr, Shah N, Madduri D, Berdeja J, Lonial S, et al. Idecabtagene vicleucel in relapsed and refractory multiple myeloma. N Engl J Med. 2021;384:705–16. 33626253 10.1056/NEJMoa2024850

[3] Martin T, Usmani SZ, Berdeja JG, Agha M, Cohen AD, Hari P, et al. Ciltacabtagene autoleucel, an anti-B-cell maturation antigen chimeric antigen receptor T-cell therapy, for relapsed/refractory multiple myeloma: CARTITUDE-1 2-year follow-up. J Clin Oncol. 2023;41:1265–74. 35658469 10.1200/JCO.22.00842 PMC9937098

[4] Berdeja JG, Madduri D, Usmani SZ, Jakubowiak A, Agha M, Cohen AD, et al. Ciltacabtagene autoleucel, a B-cell maturation antigen-directed chimeric antigen receptor T-cell therapy in patients with relapsed or refractory multiple myeloma (CARTITUDE-1): a phase 1b/2 open-label study. Lancet. 2021;398:314–24. 34175021 10.1016/S0140-6736(21)00933-8

[5] Rodriguez-Otero P, Ailawadhi S, Arnulf B, Patel K, Cavo M, Nooka AK, et al. Ide-cel or standard regimens in relapsed and refractory multiple myeloma. N Engl J Med. 2023;388:1002–14. 36762851 10.1056/NEJMoa2213614

[6] Moreau P, van de Donk N, Delforge M, Einsele H, De Stefano V, Perrot A, et al. Comparative efficacy of teclistamab versus current treatments in real-world clinical practice in the prospective locommotion study in patients with triple-class-exposed relapsed and/or refractory multiple myeloma. Adv Ther. 2023;40:2412–25. 36961654 10.1007/s12325-023-02480-7 PMC10129954

[7] Bahlis NJ, Costello CL, Raje NS, Levy MY, Dholaria B, Solh M, et al. Elranatamab in relapsed or refractory multiple myeloma: the MagnetisMM-1 phase 1 trial. Nat Med. 2023;29:2570–6. 37783970 10.1038/s41591-023-02589-w PMC10579053

[8] Bumma N, Richter J, Jagannath S, Lee HC, Hoffman JE, Suvannasankha A, et al. Linvoseltamab for treatment of relapsed/refractory multiple myeloma. J Clin Oncol. 2024;42:2702–12. 38879802 10.1200/JCO.24.01008 PMC11272139

[9] Seckinger A, Delgado JA, Moser S, Moreno L, Neuber B, Grab A, et al. Target expression, generation, preclinical activity, and pharmacokinetics of the BCMA-T cell bispecific antibody EM801 for multiple myeloma treatment. Cancer Cell. 2017;31:396–410. 28262554 10.1016/j.ccell.2017.02.002

[10] van der Vuurst, de Vries AR, Boss I, Zabaleta A, Moreno L, Adams P, et al. CC-93269, a 2+1 T cell engager targeting B cell maturation antigen and CD3ε, shows antitumor activity in multiple myeloma preclinical models. Hemasphere. 2020;4:S198.

[11] Bar N, Mateos MV, Ribas P, Hansson M, Paris L, Hofmeister CC, et al. Alnuctamab (ALNUC; BMS-986349; CC-93269), a 2+1 B-cell maturation antigen (BCMA) × CD3 T-cell engager (TCE), administered subcutaneously (SC) in patients (Pts) with relapsed/refractory multiple myeloma (RRMM): updated results from a phase 1 first-in-human clinical study. Blood. 2023;142:2011.

[12] Wong SW, Bar N, Paris L, Hofmeister CC, Hansson M, Santoro A, et al. Alnuctamab (ALNUC; BMS-986349; CC-93269), a B-cell maturation antigen (BCMA) x CD3 T-cell engager (TCE), in patients (pts) with relapsed/refractory multiple myeloma (RRMM): results from a phase 1 first-in-human clinical study. Blood. 2022;140:400–2.

[13] Lee DW, Gardner R, Porter DL, Louis CU, Ahmed N, Jensen M, et al. Current concepts in the diagnosis and management of cytokine release syndrome. Blood. 2014;124:188–95. 24876563 10.1182/blood-2014-05-552729 PMC4093680

[14] Kumar S, Paiva B, Anderson KC, Durie B, Landgren O, Moreau P, et al. International Myeloma Working Group consensus criteria for response and minimal residual disease assessment in multiple myeloma. Lancet Oncol. 2016;17:e328–e46. 27511158 10.1016/S1470-2045(16)30206-6

[15] Kiesel B, Osawa M, Masilamani M, Bar M, Hsu K, Godwin C, et al. Informing the recommended phase III dose of alnuctamab, a CD3× BCMA T-cell engager, using population pharmacokinetics and exposure–response analysis. Clin Pharmacol Ther. 2024;116:866–74. 38938115 10.1002/cpt.3353

[16] Pappa CA, Tsirakis G, Samiotakis P, Tsigaridaki M, Alegakis A, Goulidaki N, et al. Serum levels of angiopoietin-2 are associated with the growth of multiple myeloma. Cancer Invest. 2013;31:385–9. 23758184 10.3109/07357907.2013.800093

[17] Shimabukuro-Vornhagen A, Gödel P, Subklewe M, Stemmler HJ, Schlößer HA, Schlaak M, et al. Cytokine release syndrome. J Immunother Cancer. 2018;6:56. 29907163 10.1186/s40425-018-0343-9 PMC6003181

[18] Mazahreh F, Mazahreh L, Schinke C, Thanendrarajan S, Zangari M, Shaughnessy JD, et al. Risk of infections associated with the use of bispecific antibodies in multiple myeloma: a pooled analysis. Blood Adv. 2023;7:3069–74. 36857755 10.1182/bloodadvances.2022009435 PMC10331406

[19] Nath K, Shekarkhand T, Costa BA, Nemirovsky D, Derkach A, Nishimura N, et al. Multiple myeloma: clinical and epidemiological. Blood. 2023;142:1957–9.

[20] Moreau P, Garfall AL, van de Donk N, Nahi H, San-Miguel JF, Oriol A, et al. Teclistamab in relapsed or refractory multiple myeloma. N Engl J Med. 2022;387:495–505. 35661166 10.1056/NEJMoa2203478 PMC10587778

[21] Lee H, Ahn S, Maity R, Leblay N, Ziccheddu B, Truger M, et al. Mechanisms of antigen escape from BCMA- or GPRC5D-targeted immunotherapies in multiple myeloma. Nat Med. 2023;29:2295–306. 37653344 10.1038/s41591-023-02491-5 PMC10504087

