# Supplementary material for: Alnuctamab, a bivalent B-cell maturation antigen-targeting T cell engager for patients with relapsed or refractory multiple myeloma: results from a phase 1, first-in-human study
Source: Leukemia. 2026 Jan 7;40(3):481–90. doi: 10.1038/s41375-025-02841-x (PMC12960249; doi:10.1038/s41375-025-02841-x)
Supplement: Supplementary file 1 — Supplement [file 41375_2025_2841_MOESM1_ESM.docx]

Supplementary Figure 1. CONSORT diagram for the ALNUC SC and IV study arms (A) and for the target dose cohorts within the SC arm (B)


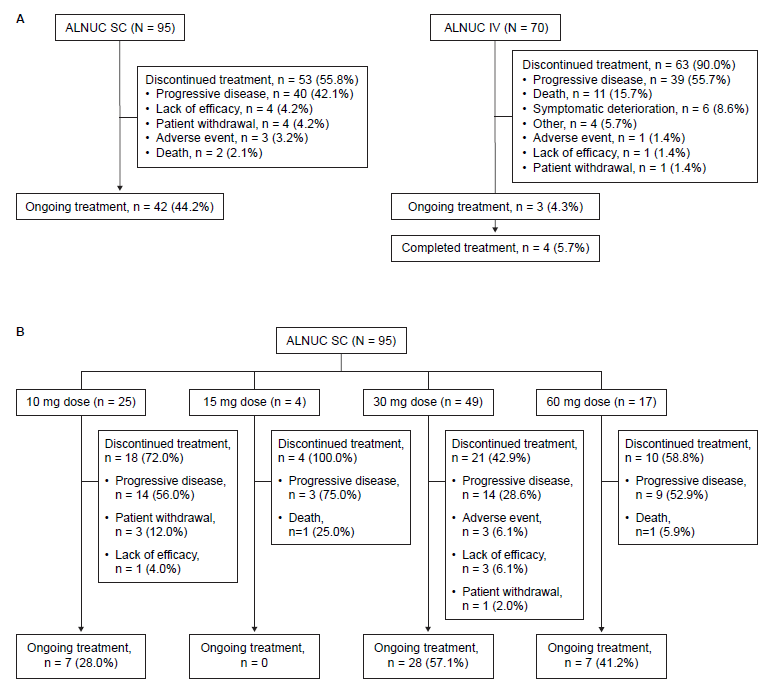


ALNUC, alnuctamab; IV, intravenous; SC, subcutaneous.


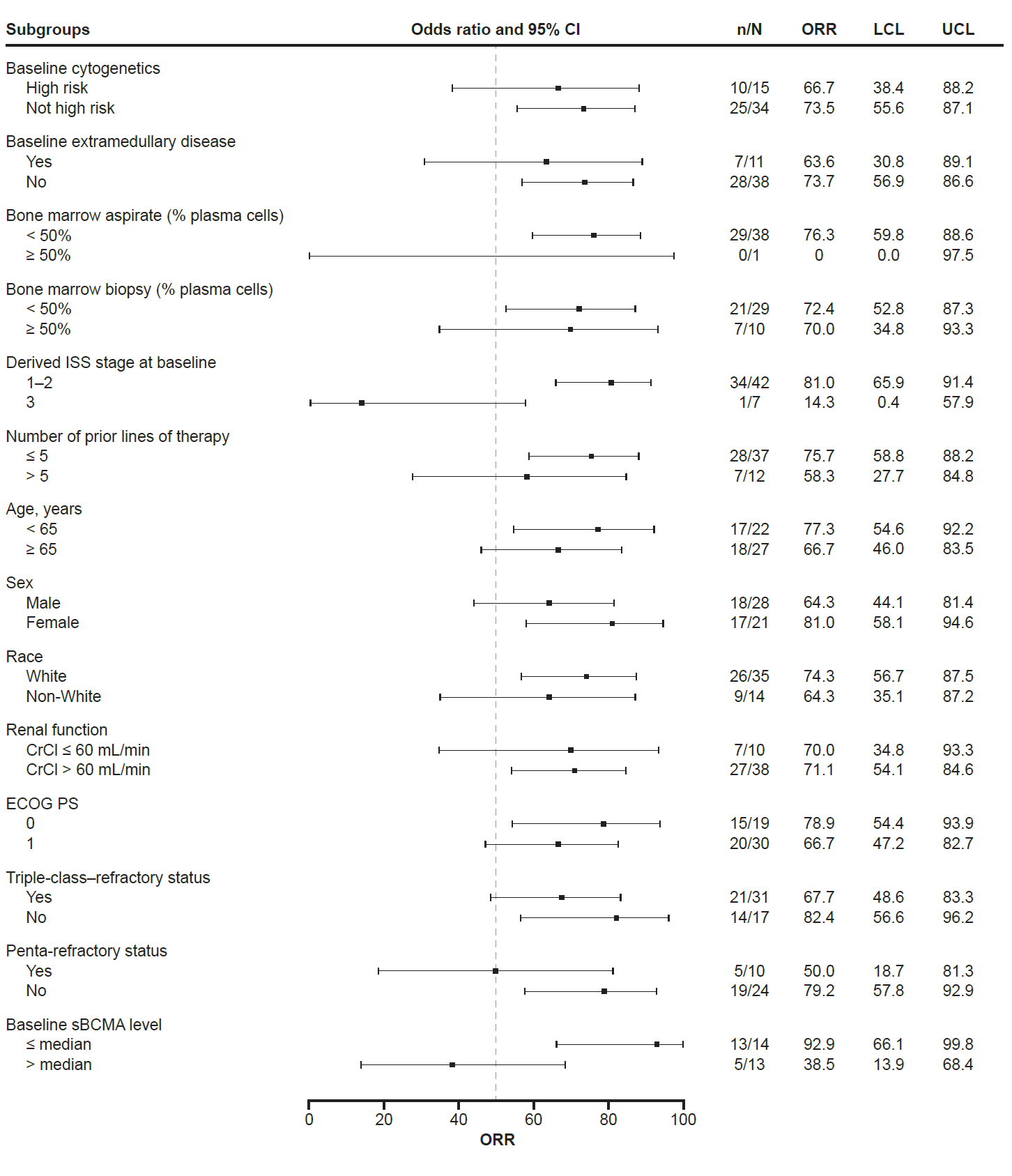
Supplementary Figure 2. ORR subgroup analysis for ALNUC SC

ALNUC, alnuctamab CL, confidence limit; CrCl, creatinine clearance; ECOG PS, ECOG performance status; ISS, international staging system; LCL, lower confidence limit; ORR, overall response rate; sBCMA, soluble B-cell maturation antigen; UPL, upper confidence limit.


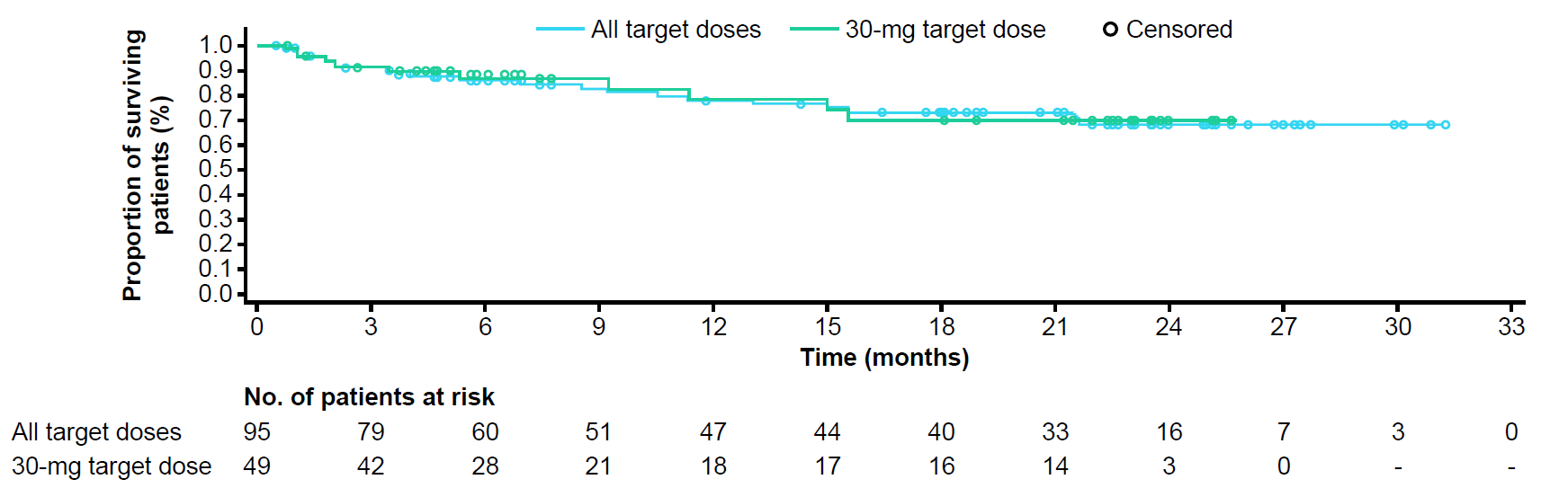
Supplementary Figure 3. OS for ALNUC SC

ALNUC, alnuctamab; OS, overall survival; SC, subcutaneous.


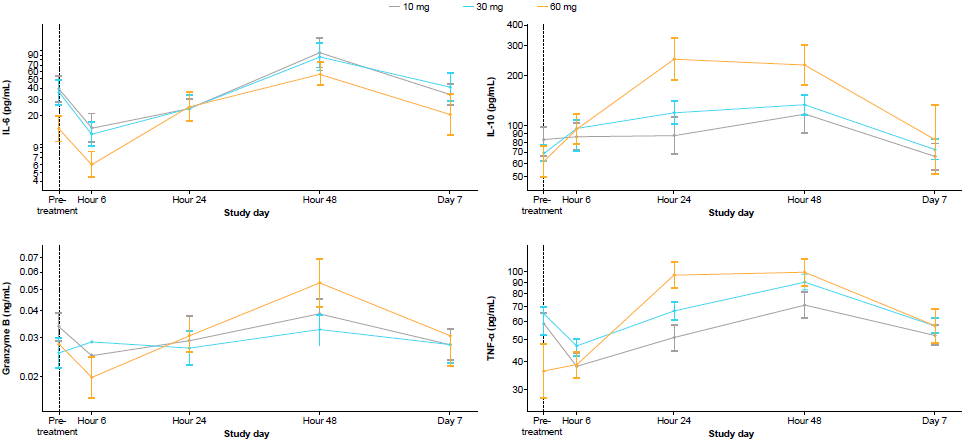
Supplementary Figure 4. Soluble factor induction over time, following the target (third) dose of ALNUC SC

ALNUC, alnuctamab; IL, interleukin; TNF-α, tumor necrosis factor alpha.

Supplementary Figure 5. Serum concentrations over time for selected T-cell activation factors, cytokines, and disease-associated factors


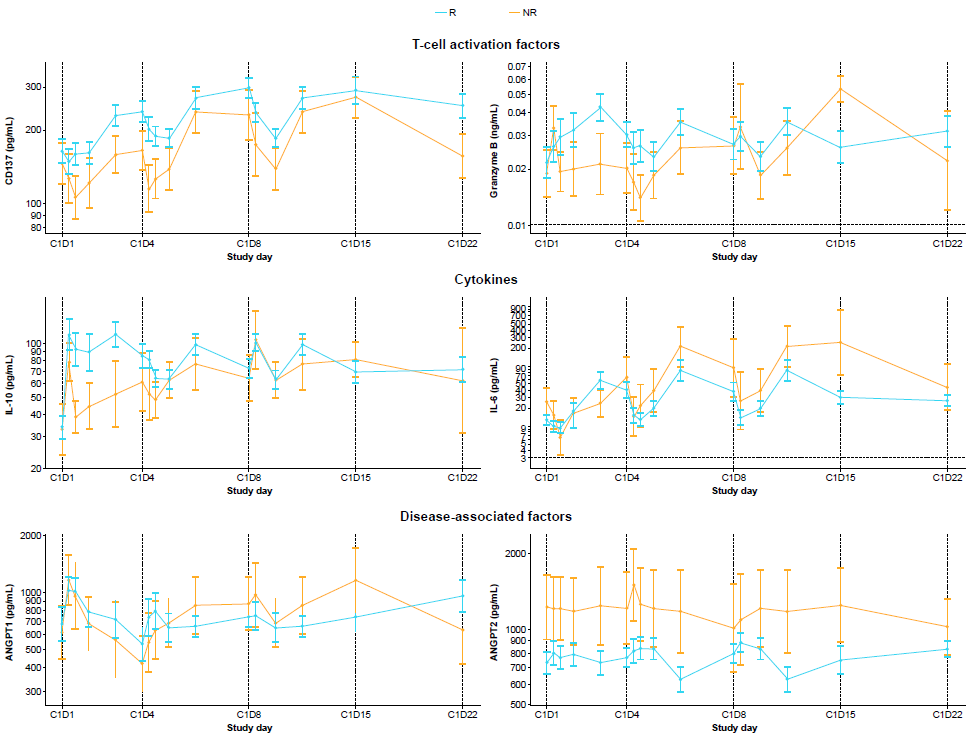


ANGPT1, angiopoietin-1; ANGPT2, angiopoietin-2; C, cycle; D, day; IL, interleukin; NR, non-responder; R, responder.

Supplementary Figure 6. B-cell clearance over time in ALNUC SC cohorts (responders^a^ [n = 56] and non-responders [n = 25])


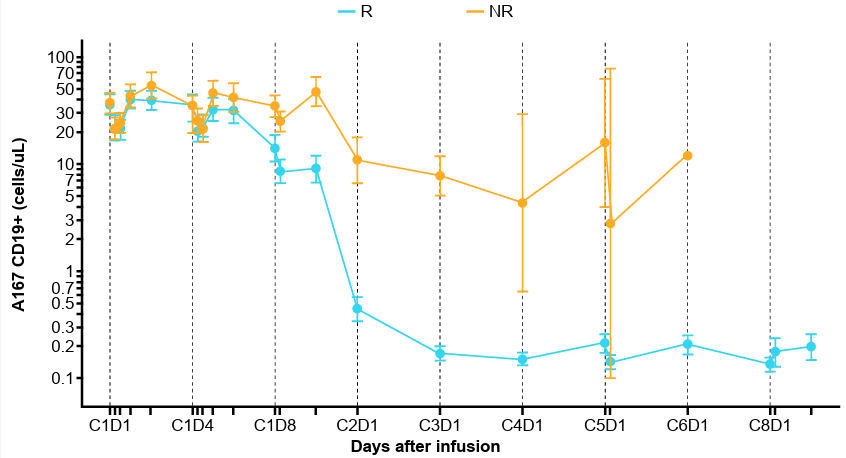


^a^Includes patients with a BOR of sCR, CR, VGPR, or PR.
ALNUC, alnuctamab; BOR, best overall response; C, cycle; CR, complete response; D, day; NR, non-responder; PR, partial response; R, responder; sCR, stringent complete response; VGPR, very good partial response.

Supplementary Figure 7. Exposure-adjusted rate of infections in ALNUC SC cohorts (30 mg and all target doses)


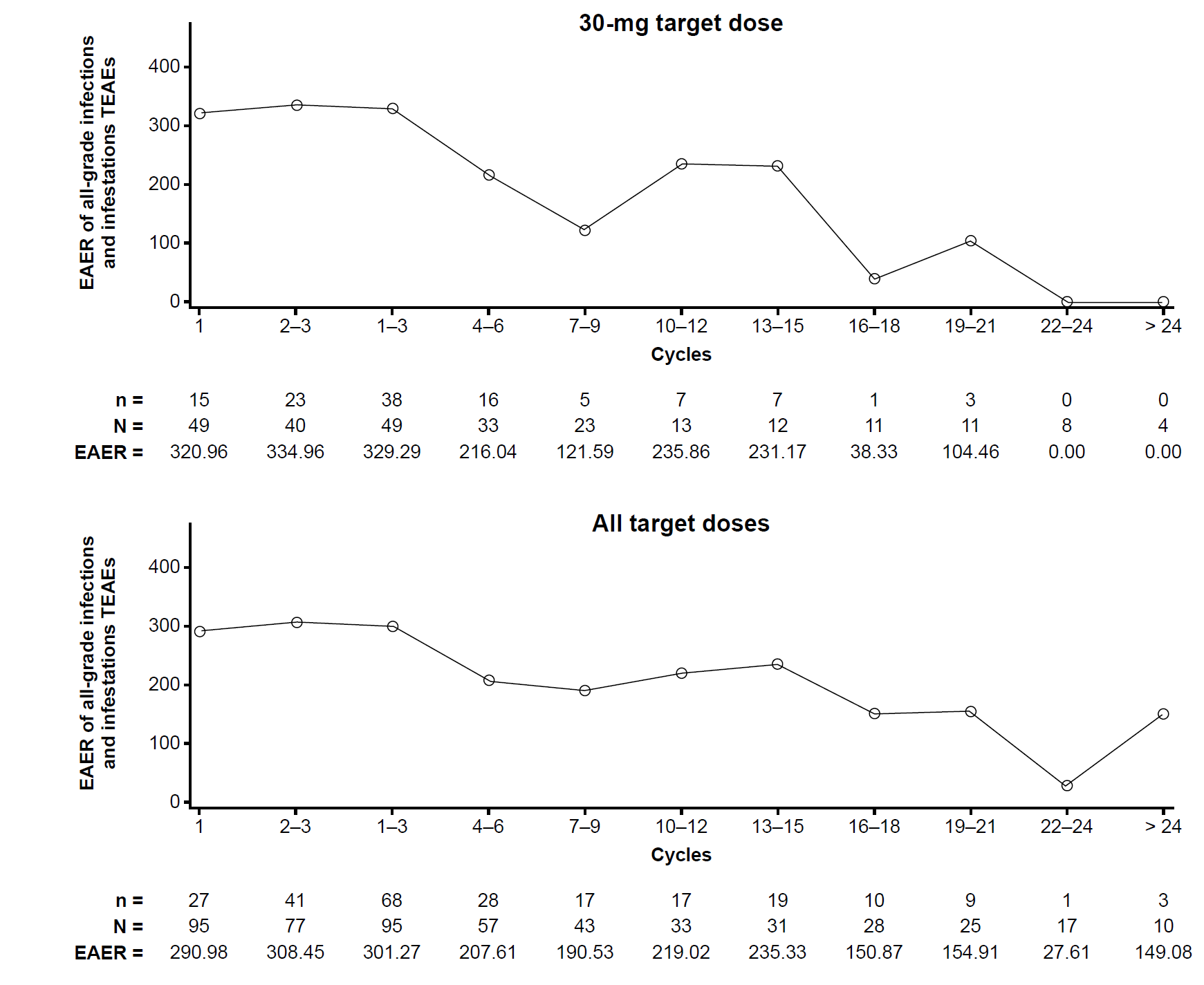


ALNUC, alnuctamab; EAER, exposure-adjusted event rate per 100 person-years (i.e., 100*n/[t/365.25], where n = number of events, and t = the sum of treatment duration in days over all treated patients);TEAE, treatment-emergent adverse event.

Supplementary Table 1. Baseline characteristics of the ALNUC IV cohort

| **Patient characteristics** | **All target doses (n = 70)** |
| --- | --- |
| **Median age, years (range)** | 64 (42–78) |
| **Sex, n (%)** |  |
| Male | 45 (64.3) |
| Female | 25 (35.7) |
| **Ethnicity, n (%)** |  |
| White | 54 (77.1) |
| African American/Black | 8 (11.4) |
| Asian | 1 (1.4) |
| American Indian or Alaska Native | 1 (1.4) |
| Native Hawaiian or Other Pacific Islander | 1 (1.4) |
| Other | 3 (4.3) |
| Not reported | 2 (2.9) |
| **ECOG performance status, n (%)** |  |
| 0 | 19 (27.1) |
| 1 | 51 (72.9) |
| **Median time since initial diagnosis, years (range)** | 5.01 (1.2–29.1) |
| **Derived ISS stage^a^** |  |
| I | 18 (25.7) |
| II | 27 (38.6) |
| III | 23 (32.9) |
| Missing | 2 (2.9) |
| **High-risk cytogenetics, n (%)^b^** | 23 (32.9) |
| **EMP, n ‘yes’ (%)** | 22 (31.4) |
| **Median BMPC, % (range)** |  |
| Aspirate | 8 (0–100) |
| Biopsy | 20 (0–100) |
| **Prior treatment history** | **All target doses (n = 70)** |
| **Median no. of prior therapies, n (range)** | 5 (3–14) |
| **Refractory to last therapy, n (%)** | 68 (97.1) |
| **Prior anti-CD38, n (%)** | 69 (98.6) |
| **Prior PI, n (%)** | 70 (100) |
| **Prior IMiD, n (%)** | 70 (100) |
| **Prior triple-class–exposed, n (%)^c^** | 48 (68.6) |
| **Prior penta-drug–exposed, n (%)^d^** | 49 (70.0) |
| **Refractory, n (%)** |  |
| Triple | 15 (21.4) |
| Penta | 16 (22.9) |
| PI | 58 (82.9) |
| IMID | 58 (82.9) |
| CD38 | 63 (90.0) |
| **Stem cell transplantation, n (%)** |  |
| Autologous | 56 (80) |
| Allogeneic | 4 (5.7) |

^a^Based on baseline ꞵ2m (central lab) and albumin (local lab).  ^b^At least one of the following: 17p del, t(4:14), t(14:16), and 1q21 amp in central lab specimen.  ^c^Patients with prior bortezomib, carfilzomib, pomalidomide, lenalidomide and anti-CD38.  ^d^Patients with ≥ 2 prior IMiD agents (i.e., lenalidomide and pomalidomide, excluding thalidomide), ≥2 prior PIs (at least two of bortezomib, carfilzomib, and ixazomib) and ≥ 1 prior anti-CD38.
ALNUC; alnuctamab; ꞵ2m, ꞵ2-microglobulin; BMPC, bone marrow plasma cells; ECOG, Eastern Cooperative Oncology Group; EMP, extramedullary plasmacytoma; IMiD, immunomodulatory imide drug; ISS, international staging system; IV; intravenous; PI, proteasome inhibitor.

Supplementary Table 2. Summary of TEAEs reported in ≥ 10% of patients^a^ treated with ALNUC IV

| **TEAEs, n (%)** | **All target doses (n = 70)** | |
| --- | --- | --- |
|  | **All grade** | **Grade 3/4** |
| **Patients with ≥ 1 TEAE** | 70 (100.0) | 62 (88.6) |
| **Blood and lymphatic system disorders** | 59 (84.3) | 53 (75.7) |
| Neutropenia | 42 (60.0) | 40 (57.1) |
| Anemia | 38 (54.3) | 28 (40.0) |
| Thrombocytopenia | 36 (51.4) | 23 (32.9) |
| Leukopenia | 7 (10.0) | 4 (5.7) |
| **Nonhematologic TEAEs** |  |  |
| Cytokine release syndrome | 53 (75.7) | 5 (7.1) |
| Diarrhea | 20 (28.6) | 4 (5.7) |
| Cough | 19 (27.1) | 0 |
| Nausea | 17 (24.3) | 0 |
| Vomiting | 15 (21.4) | 0 |
| Constipation | 14 (20.0) | 0 |
| Back pain | 14 (20.0) | 3 (4.3) |
| Fatigue | 13 (18.6) | 0 |
| Pyrexia | 13 (18.6) | 3 (4.3) |
| Upper respiratory tract infection | 12 (17.1) | 1 (1.4) |
| Headache | 12 (17.1) | 1 (1.4) |
| Hypokalemia | 11 (15.7) | 3 (4.3) |
| Pneumonia | 11 (15.7) | 10 (14.3) |
| Arthralgia | 10 (14.3) | 1 (1.4) |
| Asthenia | 10 (14.3) | 2 (2.9) |
| Pain in extremity | 9 (12.9) | 2 (2.9) |
| Blood creatinine increased | 9 (12.9) | 1 (1.4) |
| Urinary tract infection | 9 (12.9) | 4 (5.7) |
| General physical health deterioration | 9 (12.9) | 5 (7.1) |
| Hypomagnesaemia | 8 (11.4) | 0 |
| Hypophosphatemia | 8 (11.4) | 1 (1.4) |
| Dyspnea | 8 (11.4) | 0 |
| Infusion related reaction | 8 (11.4) | 0 |
| Hypogammaglobulinemia | 8 (11.4) | 3 (4.3) |
| Bone pain | 7 (10.0) | 0 |
| Hypocalcemia | 7 (10.0) | 0 |

^a^Out of 70 patients who received any target dose of ALNUC IV and experienced any-grade TEAE.
ALNUC; alnuctamab; TEAE, treatment-emergent adverse event; IV, intravenous.

Supplementary Table 3. Summary of serious TEAEs reported in at least 2 patients^a^ treated with ALNUC SC

| **TEAEs, n (%)** | **All target doses (n = 95)** | **30-mg target dose (n = 49)** |
| --- | --- | --- |
| **Patients with ≥ 1 TEAE** | 36 (37.9) | 18 (36.7) |
| **Blood and lymphatic system disorders** |  |  |
| Febrile neutropenia | 4 (4.2) | 2 (4.1) |
| **Non-hematologic TEAEs** |  |  |
| General physical health deterioration | 5 (5.3) | 3 (6.1) |
| Cytokine release syndrome | 4 (4.2) | 1 (2.0) |
| COVID-19 | 3 (3.2) | 1 (2.0) |
| Pneumonia | 3 (3.2) | 2 (4.1) |
| Pathological fracture | 3 (3.2) | 0 |
| COVID-19 pneumonia | 2 (2.1) | 1 (2.0) |
| Sepsis | 2 (2.1) | 0 |

^a^Out of 95 patients who received any target dose of ALNUC SC.
ALNUC; alnuctamab; SC, subcutaneous; TEAE, treatment-emergent adverse event.

Supplementary Table 4. Number of CRS events by duration in patients treated with ALNUC SC

| **CRS events, n (%)** | **All target doses, total no. of CRS events (n = 84)** | **30-mg target dose, total no. of CRS events,  (n = 47)** |
| --- | --- | --- |
| **Duration, days** |  |  |
| 1 | 39 (46.4) | 29 (61.7) |
| 2 | 24 (28.6) | 11 (23.4) |
| 3 | 9 (10.7) | 4 (8.5) |
| 4 | 2 (2.4) | 0 |
| 5 | 2 (2.4) | 1 (2.1) |
| 6 | 3 (3.6) | 1 (2.1 |
| 7 | 1 (1.2) | 0 |
| 8 | 1 (1.2) | 0 |
| 9 | 2 (2.4) | 1 (2.1) |
| 10 | 0 | 0 |
| 11 | 1 (1.2) | 0 |

ALNUC; alnuctamab; CRS, cytokine release syndrome; SC, subcutaneous.

Supplementary Table 5. Grade 3/4 and serious treatment-emergent infections with ALNUC SC^a^

| **Grade 3/4 treatment-emergent infections, n (%)** | **All target doses**  **(n = 95)** | **30-mg target dose**  **(n = 49)** |
| --- | --- | --- |
| Pneumonia | 3 (3.2) | 2 (4.1) |
| COVID-19 | 2 (2.1) | 1 (2.0) |
| Sepsis | 2 (2.1) | 0 |
| Arthritis bacterial | 1 (1.1) | 0 |
| COVID-19 pneumonia | 1 (1.1) | 0 |
| Clostridium difficile colitis | 1 (1.1) | 0 |
| Influenza | 1 (1.1) | 0 |
| Pseudomonal bacteremia | 1 (1.1) | 0 |
| Respiratory tract infection | 1 (1.1) | 1 (2.0) |
| Salmonellosis | 1 (1.1) | 1 (2.0) |
| Streptococcal bacteremia | 1 (1.1) | 0 |
| Urinary tract infection | 1 (1.1) | 0 |
| **Serious treatment-emergent infections, n (%)** | **All target doses**  **(n = 95)** | **30-mg target dose**  **(n = 49)** |
| COVID-19 | 3 (3.2) | 1 (2.0) |
| Pneumonia | 3 (3.2) | 2 (4.1) |
| COVID-19 pneumonia | 2 (2.1) | 1 (2.0) |
| Sepsis | 3 (3.2) | 0 |
| Arthritis bacterial | 1 (1.1) | 0 |
| Bacteremia | 1 (1.1) | 0 |
| Clostridium difficile colitis | 1 (1.1) | 0 |
| Device related infection | 1 (1.1) | 1 (2.0) |
| Influenza | 1 (1.1) | 0 |
| Medical device site joint infection | 1 (1.1) | 0 |
| Pseudomonal bacteremia | 1 (1.1) | 0 |
| Streptococcal bacteremia | 1 (1.1) | 0 |

^a^Patients may have been counted more than once if they experienced multiple treatment-emergent infections.
ALNUC, alnuctamab; SC, subcutaneous.

Supplementary Table 6. Summary of deaths for ALNUC SC

|  | **All target doses (n = 95)** | **30-mg target dose (n = 49)** |
| --- | --- | --- |
| **Overall number of deaths, n (%)** | 23 (24.2) | 10 (20.4) |
| **Deaths within 35 days^a^ after last dose of study drug, n (%)** | 7 (7.4) | 3 (6.1) |
| Death from malignant disease under study, or complication due to malignant disease under study | 3 (3.2) | 2 (4.1) |
| Death from AE (not otherwise specified) | 3 (3.2) | 0 |
| Missing | 1 (1.1) | 1 (2.0) |
| **Deaths more than 35 days^a^ after last dose of study drug, n (%)** | 16 (16.8) | 7 (14.3) |
| Death from malignant disease under study, or complication due to malignant disease under study | 10 (10.5) | 5 (10.2) |
| Death from AE (not otherwise specified) | 3 (3.2) | 1 (2.0) |
| Death from other cause^b^ | 1 (1.1) | 1 (2.0) |
| Missing | 2 (2.1) | 0 |

^a^Safety follow-up period.
^b^The cause of death was cardiac arrest.
AE, adverse event; ALNUC; alnuctamab; SC, subcutaneous.

Supplementary Table 7. Summary of key efficacy endpoints for ALNUC IV

| **Patient response rates** | **All target doses (n = 70)** |
| --- | --- |
| **ORR, n (%)** | 28 (40.0) |
| sCR n (%) | 12 (17.1) |
| CR | 5 (7.1) |
| VGPR | 5 (7.1) |
| PR | 5 (7.1) |
| **Median DOR, months (95% CI)** | 25.9 (10.6–NE) |
| **Median PFS, months (95% CI)** | 3.1 (1.9–5.5) |
| **Median OS, months (95% CI)** | 15.9 (8.0–28.4) |

ALNUC, alnuctamab; CR, complete response; DOR, duration of response; IV, intravenous; ORR, objective response rate; NE, not evaluable; OS, overall survival; PFS, progression-free survival; PR, partial response; sCR, stringent complete response; VGPR, very good partial response.
